# Painless nondiabetic lumbosacral radiculoplexus neuropathy with complete recovery: a case report

**DOI:** 10.1186/s13256-023-04227-y

**Published:** 2023-11-21

**Authors:** Zhibin Tan, Wai Dic Foong, You-Jiang Tan

**Affiliations:** 1 National Neuroscience Institute (Singapore General Hospital Campus), Hospital Drive 1 Outram Road, Singapore, 169608 Singapore; 2 Neuroscience Academic Clinical Programme, Duke-NUS Medical School, Singapore, Singapore

**Keywords:** Amyotrophy, Neurology, Clinical, Localization, Painless, Nondiabetic, Immunoglobulin, Immunotherapy, Recovery, Case report

## Abstract

**Background:**

Lumbosacral radiculoplexus neuropathy, also known as amyotrophy, is an uncommon monophasic disorder characterized by inflammation of the lumbosacral nerve roots and plexuses. Lumbosacral radiculoplexus neuropathy is usually associated with diabetes mellitus, is typically painful at presentation, and often associated with long-term residual neurologic deficits. We report a case of painless, nondiabetic lumbosacral radiculoplexus neuropathy in a young Chinese woman, who made a full recovery after treatment with intravenous immunoglobulin, adding an atypical case to the scarce literature on lumbosacral radiculoplexus neuropathy.

**Case presentation:**

A 35-year-old Chinese woman presented to our emergency department with 1-week history of painless left lower limb weakness and numbness. Examination revealed weakness confined to the left lower limb but spanning various nerves and myotomes, with abnormal sensation. Clinical localization to the lumbosacral plexus was supported by neurodiagnostic tests, and magnetic resonance imaging of the lumbosacral plexus showed that the nerve roots were also involved. After treatment with intravenous immunoglobulin for nondiabetic lumbosacral radiculoplexus neuropathy, the patient had a full recovery.

**Conclusion:**

Our patient’s case highlights that lumbosacral radiculoplexus neuropathy, an already rare disorder, can occur in the absence of diabetes mellitus and pain, making it even harder to recognize. A systematic and meticulous clinical approach, supported by intelligent selection of adjunctive tests, is required for localization and diagnosis. With an accurate diagnosis, our case also demonstrates that appropriate and prompt treatment can lead to complete recovery, despite previous reports suggesting a high prevalence of long-term residual deficits after lumbosacral radiculoplexus neuropathy.

## Introduction

Lumbosacral radiculoplexus neuropathy (LRPN), also known as amyotrophy, is an uncommon monophasic disorder characterized by inflammation of the lumbosacral nerve roots and/or plexuses, usually associated with diabetes mellitus [1–3]. The disorder is typically associated with pain and long-term residual deficits [7, 8]. Herein, we aim to raise awareness of this rare disorder and its heterogeneous presentation by reporting an unusual case of painless, nondiabetic LRPN in a young Chinese lady with full recovery after treatment with intravenous immunoglobulin (IVIg).

## Case presentation

A 35-year-old Chinese woman with no significant past medical history, family history, or medication history was admitted via our emergency department with a 1-week history of gradually worsening left lower limb weakness and numbness. She was without pain, and had no significant past medical history or recent illnesses. On examination, her left-sided ankle deep-tendon reflex was absent, and there was diffuse weakness (modified medical research council scale grade 4) of hip extension, hip adduction, knee extension, knee flexion, ankle plantar flexion, and ankle dorsiflexion in the left lower limb. Sensory testing demonstrated diminished proprioception and pain perception in the left lower limb, distal to the mid-calf. The rest of the neurological examination was unremarkable. Her gait was slow and cautious, but otherwise normal despite her subjective complaints of gait difficulty.

**Fig. 1:**
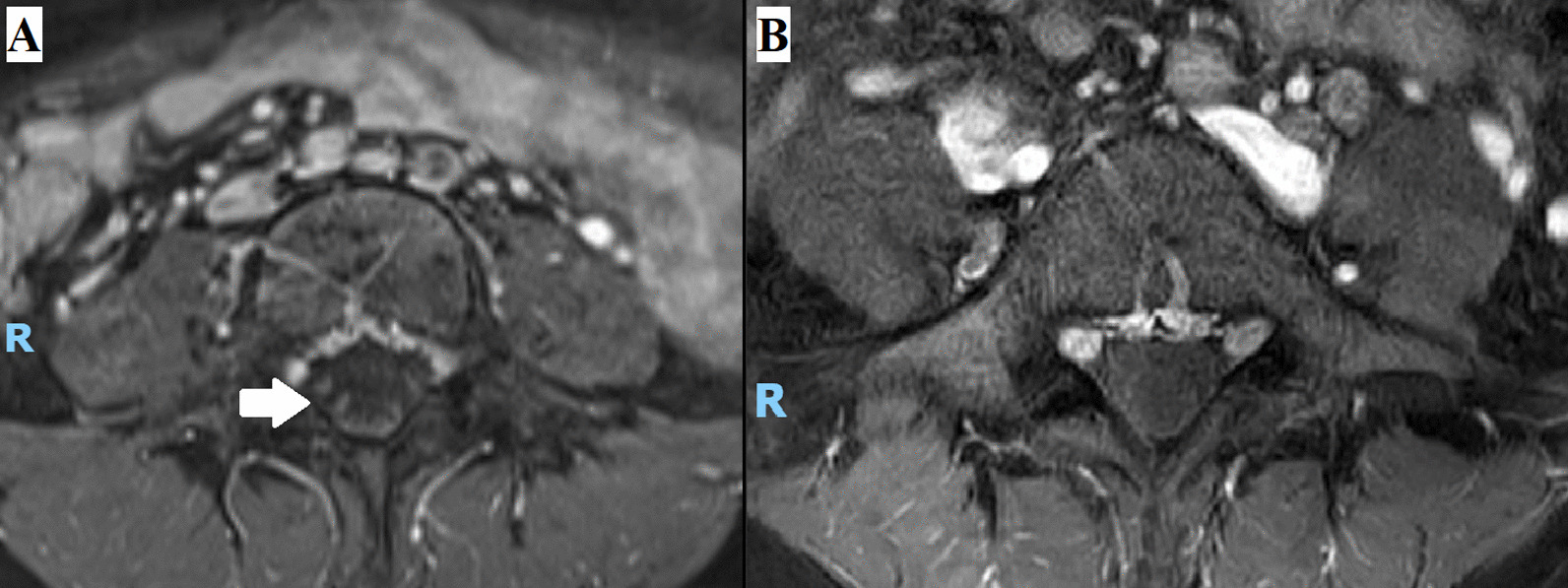
MRI of the lumbar spine. **A** Axial projection, T1 with gadolinium contrast on the eighth day of symptoms, showing enhancement of the cauda equina nerve roots (arrow) at the L4 vertebral level. **B** Axial projection, T1 with gadolinium contrast on the 18th week from symptom onset, showing resolution of the nerve root enhancement

A nerve conduction study (NCS) and electromyography (EMG) performed eight days from symptom onset suggested a plexopathy. Specifically, there was an asymmetrically absent left-sided tibial H-reflex, diminished left saphenous sensory response of 1.93 μV compared with a right saphenous sensory response of 4.2 μV, and reduced recruitment on EMG of the left-sided vastus lateralis, gastrocnemius, and tibialis anterior muscles. Gadolinium-enhanced magnetic resonance imaging (MRI) of the lumbosacral plexuses and lumbar spine demonstrated enhancement of the cauda equina nerve roots bilaterally, sparing the lumbosacral plexuses (Fig. 1). A lumbar puncture was performed 12 days from symptom onset, showing a normal opening pressure of 12 cm H_2_O. Importantly, cerebrospinal fluid (CSF) examination revealed cytoalbuminologic dissociation (CSF protein 1.03 g/L, with a CSF white blood cell count of 1/μL) but was otherwise negative for oligoclonal bands. Microbiological and autoimmune assays performed on blood and CSF samples were unyielding (Table 1). Computed tomography (CT) of her neck, thorax, abdomen, and pelvis showed no suspicious masses.

**Table 1:** Summary of blood and cerebrospinal fluid investigations. All negative or normal

Tests on serum/blood	
Microbiology	HIV, hepatitis B, hepatitis C, EBV
Autoimmune antibodies	ANA, ANCA, antidouble stranded DNA antibody, Smith, ribonucleoprotein, Ro, La, Scl 70, Jo-1
Onconeural antibodies	Hu, Yo, Ri, CRMP5, amphiphysin, PNMA2, recoverin, SOX1, titin, zic4, GAD65, Tr
IgG ganglioside antibodies	MAG, GM1, GM2, GD1a, GD1b, GQ1b
IgM ganglioside antibodies	MAG, GM1, GM2, GD1a, GD1b, GQ1b
Tests on CSF	
Microbiological tests	Gram stain, culture VDRL PCR tests (*Escherichia coli*, *Haemophilus influenzae*, *Listeria*, *Neisseria meningitidis*, *Streptococcus agalactiae*, *Streptococcus pneumoniae*, CMV, enterovirus, HSV1, HSV2, human parechovirus, VZV, *Cryptococcus*)
Inflammatory/demyelinating	Oligoclonal bands (with paired serum sample)
Malignancy	Flow cytometry Cytology (no malignant cells)

*ANA* antinuclear antibody, *ANCA* antineutrophil cytoplasmic antibody, *CMV* cytomegalovirus, *CRMP5* collapsing response mediator protein 5, *CSF* cerebrospinal fluid, *EBV* Epstein–Barr virus, *GAD65* glutamic acid decarboxylase 65-kilodalton isoform, *HIV* human immunodeficiency virus, *HSV* herpes simplex virus, *MAG* myelin-associated glycoprotein, *PCR* polymerase chain reaction, *PNMA2* paraneoplastic antigen Ma2, *SOX1* Sry-like high mobility group box 1, *VDRL* venereal disease research laboratory, *VZV* varicella zoster virus

The patient was diagnosed with nondiabetic LRPN and was treated with IVIg at 2 g/kg divided over 5 days (13–17 days from symptom onset). The patient was reviewed by a physiotherapist once during her hospital stay, but did not require further rehabilitation. Other than a brief 3-day period of postdural puncture headache, which resolved with bed rest and hydration, she tolerated the evaluation and treatment well. She was discharged upon resolution of headache (18 days from symptom onset) with no change in clinical examination findings. On subsequent outpatient follow-up, she reported that her symptoms gradually resolved over the 2 weeks succeeding her discharge from hospital, and a subsequent MRI scan of her lumbar spine performed 18 weeks after symptom onset showed resolution of the cauda equina nerve root enhancement (Fig. 1). She remained asymptomatic when reviewed 20 weeks after symptom onset, and examination was normal, demonstrating a return of her left ankle tendon reflex and full power in all limbs. One year after the episode, the patient reported by phone interview that she had no recurrence of symptoms.

## Discussion

LRPN is an uncommon, monophasic disease characterized by inflammation of multiple lumbosacral nerve roots and the lumbosacral plexuses, usually in association with diabetes mellitus [1]. While diabetic LRPN was first described in 1890, nondiabetic LRPN was more recently reported in 1981 by Evans *et al.* and appears rarer, with a postulated annual incidence rate of 1.27 per 100,000 [2, 4, 7]. The clinical features of nondiabetic LRPN are thought to be similar to diabetic LRPN [3]. In a 2019 study of 62 diabetic and nondiabetic LRPNs, Ng P.S. *et al.* reported that nearly two-thirds of cases (62.9%, 39/62) had unilateral symptoms at presentation, and unilateral deficits appeared more often among nondiabetic LRPN cases (80%, 16/20) than in diabetic LRPNs (54.8%, 23/42) [7]. In a follow-up analysis of these 62 patients, Pinto *et al.* reported that the temporal profile was hyperacute (< 24 hours to nadir) in 3.2%, rapidly progressive (1 day to 1 week) in 24.2%, and subacute to chronic (> 1 month) in 66.1% [8]. Our patient’s symptoms represented a case of rapidly progressive LRPN, reaching a nadir after 1 week from onset.

Our patient’s case of nondiabetic LRPN highlights important learning points.

First, we wish to raise awareness among clinicians that LRPN, known to some as amyotrophy, can occur in nondiabetic patients. Furthermore, LRPN can be painless at presentation. In the 2019 study, Ng *et al.* reported that five patients (8.1%) were without pain, amongst whom three were nondiabetic, representing the first descriptions of painless nondiabetic LRPN [4, 7]. Our patient therefore adds to this rare and recently described phenomenon, and clinicians should give due consideration to a diagnosis of nondiabetic LRPN even when there is no pain.

Secondly, a meticulous neurological examination is vital in localizing and diagnosing LRPN. Such keen clinical attention to detail is even more important when dealing with an uncommon presentation of an already rare disorder, such as a painless, nondiabetic variant of LRPN in our patient. Clinically, our patient’s weakness of left hip extension indicated involvement of the gluteus maximus and hamstring muscles, or the nerves that supply them, which are the inferior gluteal nerve (S1 myotome) and sciatic nerve (L5 and S1 myotomes) respectively [9]. Weakness of left ankle plantar flexion and dorsiflexion confirmed that both tibial and peroneal divisions of the sciatic nerve were involved, and expanded the myotomes involved to include L4 [9]. Weakness of left knee extension suggested involvement of the quadriceps femoris muscle, supplied by the femoral nerve (L2, L3, and L4 myotomes) [9]. Finally, weakness of left hip adduction further implicated the adductors of the lower limb, supplied by the obturator nerve (mainly L3 and L4 myotomes) [9]. Given the multiple nerves and myotomes involved, one should consider a plexopathy or multilevel radiculopathy to be the most likely localization. The fact that there were also sensory deficits in the left lower limb ruled out a primary neuromuscular junction disorder or focal myopathy. In addition, pin-prick sensation was diminished in the ipsilateral (left) lower limb but normal in the contralateral (right) lower limb, therefore excluding a hemispinal cord localization (that is, Brown Sequard syndrome). With the above considerations, neurodiagnostic tests were selectively performed. The finding of reduced recruitment on EMG of several left lower limb muscles confirmed that the pathology was a neuropathic rather than myopathic disorder. Importantly, as radiculopathy should not cause abnormal sensory responses, the asymmetrically diminished left saphenous sensory response indicated a plexopathy, with or without nerve root involvement [9]. Building on this localization, MRI of the lumbosacral plexus was arranged, showing enhancing nerve roots and thus suggesting that the disorder involved both nerve roots and plexus. The focal nature of presenting symptoms, mostly preserved reflexes, absence of cranial neuropathies, absence of demyelination on NCS, and negative ganglioside antibodies further made a diagnosis of Guillain Barré syndrome (GBS) unlikely. Due to the absence of specific distinguishing clinical features of LRPN, the astute clinician needs to maintain an appropriate index of suspicion even in the absence of diabetes and pain.

Our third learning point is that patients with LRPN may have bilateral enhancement on MRI scans, even when the symptoms and signs are unilateral. One possible explanation is that clinical presentation depends on the severity of inflammation, whereas the MRI findings indicate the extent of involvement without necessarily reflecting severity. This explanation is further supported by previous observations that electrophysiological tests can also demonstrate more widespread involvement than the clinical deficits suggest [3].

The final learning point is the potential value of IVIg in the treatment of patients with nondiabetic LRPN. LRPNs often result in long-term residual neurological deficits, but our patient represents a rare case of complete recovery [8]. While it remains uncertain if our patient’s excellent outcome was entirely influenced by IVIg treatment, histological evidence by preceding studies demonstrating underlying immune-mediated, inflammatory pathogenic processes behind LRPN lends reason to the administration of IVIg [3, 5]. There have also been previous case reports and uncontrolled case series demonstrating recovery in LRPNs after treatment with immunotherapy (involving varying combinations of IVIg, intravenous methylprednisolone, and oral prednisolone), providing further support [6, 10–12].

## Conclusion

While current clinical descriptions of painless, nondiabetic LRPN remain scarce, our patient’s clinical presentation, treatment, and recovery can add further to the characterization of this uncommon neurological disorder. The learning points illustrated: (1) LRPN can occur in the absence of diabetes mellitus and can be painless at presentation, (2) meticulous examination with targeted adjunctive tests is important for diagnosing LRPN, (3) imaging findings can be more extensive and bilateral even if clinical deficits are focal, and (4) immunotherapy may be useful in patients with nondiabetic LRPN, can help augment the current understanding of this uncommon disorder.

## Data Availability

Not applicable.

## Abbreviations

ANA: Antinuclear antibody
ANCA: Antineutrophil cytoplasmic antibody
CMV: Cytomegalovirus
CRMP5: Collapsing response mediator protein 5
CSF: Cerebrospinal fluid
CT: Computed tomography
EBV: Epstein–Barr virus
EMG: Electromyography
GAD65: Glutamic acid decarboxylase 65-kilodalton isoform
GBS: Guillain Barré syndrome
HIV: Human immunodeficiency virus
HSV: Herpes simplex virus
IVIg: Intravenous immunoglobulin
LRPN: Lumbosacral radiculoplexus neuropathy
MAG: Myelin-associated glycoprotein
MRI: Magnetic resonance imaging
NCS: Nerve conduction study
PCR: Polymerase chain reaction
PNMA2: Paraneoplastic antigen Ma2
SOX1: Sry-like high mobility group box 1
VDRL: Venereal disease research laboratory
VZV: Varicella zoster virus
